# EMNUSS: a deep learning framework for secondary structure annotation in cryo-EM maps

**DOI:** 10.1093/bib/bbab156

**Published:** 2021-05-05

**Authors:** Jiahua He, Sheng-You Huang

**Affiliations:** School of Physics, Huazhong University of Science and Technology, Wuhan, Hubei 430074, P. R. China; School of Physics, Huazhong University of Science and Technology, Wuhan, Hubei 430074, P. R. China

**Keywords:** cryo-electron microscopy (cryo-EM), secondary structure, deep learning, nested U-net, EM maps

## Abstract

Cryo-electron microscopy (cryo-EM) has become one of important experimental methods in structure determination. However, despite the rapid growth in the number of deposited cryo-EM maps motivated by advances in microscopy instruments and image processing algorithms, building accurate structure models for cryo-EM maps remains a challenge. Protein secondary structure information, which can be extracted from EM maps, is beneficial for cryo-EM structure modeling. Here, we present a novel secondary structure annotation framework for cryo-EM maps at both intermediate and high resolutions, named EMNUSS. EMNUSS adopts a three-dimensional (3D) nested U-net architecture to assign secondary structures for EM maps. Tested on three diverse datasets including simulated maps, middle resolution experimental maps, and high-resolution experimental maps, EMNUSS demonstrated its accuracy and robustness in identifying the secondary structures for cyro-EM maps of various resolutions. The EMNUSS program is freely available at http://huanglab.phys.hust.edu.cn/EMNUSS.

## 1 Introduction

Advances in microscopy instruments and image processing algorithms have led to an increasing number of cryo-electron microscopy (cryo-EM) maps [1–3]. The ‘resolution revolution’ in cryo-EM has paved the way for the determination of structures of previously intractable biological systems at unprecedented resolution [4–14]. However, the goal of cryo-EM is not to obtain the 3D maps but to determine the detailed atomic structures [15–25].

It is challenging to build accurate structure models for cryo-EM maps [26]. Rigid fitting and flexible fitting are commonly used methods to fit atomic structures into EM maps, but they are only possible if template structures are available. Without template structures, *de novo* modeling tools are needed to build full-atom models into EM density maps. However, the application of *de novo* modeling tools is limited because of their precarious accuracy. Owing to these difficulties, there is still a gap between the number of maps and the number of reconstructed/modeled 3D structures. As of 7 October 2020, there were 12 531 EM maps deposited in Electron Microscopy Data Resource, with only 6085 associated structures deposited in the Protein Data Bank (PDB)[27].

Protein secondary structure information, which can be extracted from maps, is demonstrated to be beneficial for both template-based fitting and *de novo* modeling [26]. PF2fit, which is a rigid fitting method, matches the detected secondary structures of the density map with the secondary structure units of the atomic model [28]. In 2017, Dou *et al.* [29] developed a flexible fitting method guided by the correspondence between the $\alpha $-helices in the cryo-EM map and those in the model. For those *de novo* modeling methods, secondary structure information plays a vital role. For example, EM-Fold places the secondary structure elements predicted from sequence to detected $\alpha $-helix density rods [16]. Iterative secondary structure refinement was also used by Pathwalking [17].

With the exponentially increasing number of deposited EM maps, various algorithms have been developed for secondary structure detection in cryo-EM maps. Traditional methods detect density regions that correspond to typical $\alpha $-helices or $\beta $-sheets [30–33]. Recently, deep learning has also been applied in the task of secondary structure classification for EM maps. In the recent decade, many attempts had been made to develop deep learning frameworks for secondary structure prediction in cryo-EM maps [34–36], including state-of-the-art Emap2sec [37] and Haruspex [38].

Emap2sec was developed for EM maps of intermediate resolutions (middle resolution), which shows promising results and provides a novel approach to structural interpretation of maps at intermediate resolution. The updated version, Emap2sec+ [39], extended the method to detect nucleic acid region in EM maps and adopted a more advanced convolutional neural network architecture, ResNet [40]. Emap2sec and Emap2sec+ perform classification for each voxel in given EM map with a stride of 2 Å. However, there are limitations with the methods. On one hand, the input of Emap2sec are cubic voxels of side length 11 Å, which is shorter than the length of an average $\alpha $-helix with 10 residues (15 Å), thus may lead to reduced accuracy. On the other hand, Emap2sec/Emap2sec+ may also be incompatible with large EM maps since these maps have astronomical number of voxels. Haruspex was developed to detect nucleotides and protein secondary structure in high-resolution EM maps using a U-Net (UNet)-style [41] deep learning architecture. The full convolutional architecture of Haruspex enables its fast and accurate prediction even on personal laptops without GPU. Nevertheless, there are also limitations for Haruspex. For convenience, Haruspex rescales the grid interval of a cryo-EM map to 1.1 Å if it is outside [1.0, 1.2] Å. However, the inconformity of grid interval will confound the feature extraction of convolution layers. In addition, Haruspex tries to predict the ‘unassigned’ regions for a given EM map. Such ‘unassigned’ regions may cause imbalance on classification and reduce the predictive power of Haruspex.

To overcome the shortcomings of existing approaches, we have introduced a novel secondary structure determination framework for cryo-EM maps at both high and intermediate resolutions, named EMNUSS. EMNUSS adopts a three-dimensional (3D) nested UNet architecture [42] that can fast and accurately predict protein secondary structures for cryo-EM maps of varied sizes. EMNUSS showed a significantly improved performance on three datasets including simulated maps, middle resolution experimental maps and high-resolution experimental maps.

## 2 Methods

### 2.1 Network architecture

**Figure 1:**
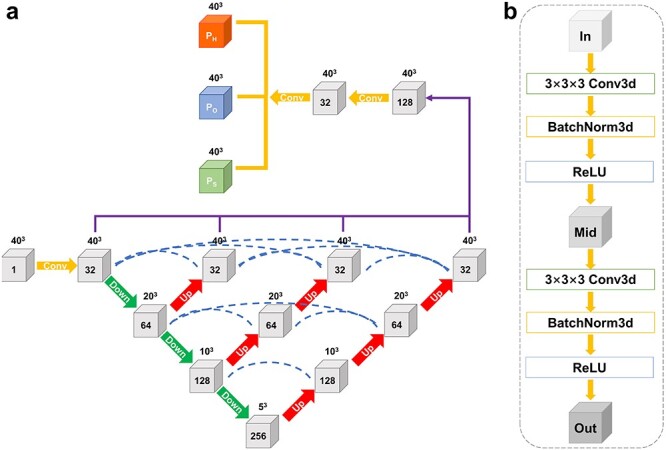
(**A**) The network architecture of EMNUSS consists of multiple interconnected layers, shown as gray cubes. Layers are connected by convolution blocks. Number of channels is labeled on each cube, and the size is labeled on the top of each cube. Yellow arrows indicate conventional convolutions, green arrows indicate 3D maxpooling with stride of 2 and red arrows indicate trilinear interpolation with zoom factor of 2. Blue dotted lines indicate skip connections. The results of the encoder–decoder subnetworks are concatenated together (purple lines) and give the secondary classification through a convolution layer. (**B**) The convolution block used to connect two layers. The combination of 3D convolution layer, 3D Batch normalization layer, and ReLU activation layer is used twice in each convolution block. ‘In’, ‘Mid’ and ‘Out’ indicate the numbers of channels. The number of ‘Mid’ channels is equal to the number of ’Out’ channels.

We adopted a nested UNet architecture to assign secondary structure for EM density maps. Figure 1 shows an overview of the EMNUSS architecture. Nested UNet (UNet++) [42] starts with an encoder subnetwork or backbone followed by a decoder subnetwork. 3D maxpooling layer with stride of 2 was used as downsampling, and trilinear interpolation layer with zoom factor of 2 was used as upsampling. Compared with the UNet used by Haruspex, UNet++ has dense skip connections on skip pathways, which improves gradient flow. Moreover, the deep supervision used in UNet++ enables model pruning and improves performance. The input of our network are density chunks with a grid interval of 1.0 Å. The chunk size was set to 40 $\times $ 40 $\times $ 40 in order to cover the secondary structure element including surrounding interaction partners. The output of our network are annotated chunks of the same size. Each voxel in the annotated chunk has three channels containing the probabilities that this voxel is close to an $\alpha $-helix residue, a $\beta $-strand residue or a coil residue.

### 2.2 Data collection

To be comparable with existing methods, the test sets of simulated maps and middle resolution maps used in Emap2sec were also used to evaluate our EMNUSS. For simulated maps, the authors of Emap2sec collected a nonredundant set of 2000 SCOPe structures [43]. we used the *e2pdb2mrc.py* program from the EMAN2 package [44] (v.2.11) to generate simulated EM maps for each structure at resolutions of 6.0 and 10.0 Å. Maps simulated from 34 structures were used as test set (Supplementary Table 1) and maps simulated from the remaining 1964 structures were used to train our EMNUSS.

For experimental maps at middle resolution, the test set of 43 experimental EM maps at 5–10 Å resolution in Emap2sec was used to evaluate EMNUSS (Supplementary Table 2). Instead of using 4-fold cross-validation like Emap2sec, we decided to build a nonredundant training set for middle resolution maps. All the EM density maps at 5–10 Å resolution that have associated PDB models were downloaded from EMDataResource. Any PDB structure and its corresponding EM map that met the following criteria were removed: (i) including nucleic acids, (ii) containing backbone atoms only, (iii) including missing chain, (iv) severe misfits between map and deposited model and (v) having any chain with over 30% sequence identity with any chain in the test set. In order to ensure criteria (iv), we calculated the cross-correlation between the deposited map and the map simulated from deposited model at the same resolution using UCSF chimera. The value of 0.65 was chosen as the threshold of cross-correlation. Afterwards, all the remaining maps were manually checked. Finally, the remaining training cases were clustered using a greedy algorithm. Two models are considered to be similar if any chain in the first model has >95% sequence identity with any chain in the second model. Case with the largest number of similar cases was chosen as a representative of the biggest cluster, and then the biggest cluster was removed from calculation. This procedure was repeated until all the cases are clustered. The final nonredundant training set consists of the representatives of each cluster. A total of 120 experimental EM maps with resolution ranging from 5.0 to 9.5 Å were retained as training set for experimental maps at middle resolution (Supplementary Table 3).

For experimental maps at high resolution, all the EM density maps with resolutions ranging from 2.0 to 4.0 Å that have associated PDB models were downloaded from the EMDB. Any PDB structure and its corresponding EM map that met the following criteria were removed: (i) including nucleic acids, (ii) containing backbone atoms only, (iii) including missing chain and (iv) severe misfits between map and deposited model. The remaining models were clustered using greedy algorithm at 30% sequence identity cutoff. A total of 468 experimental EM maps with resolution ranging from 2.0 to 4.0 Å were retained as the dataset for experimental maps at high resolution (Supplementary Table 4). The training and testing were performed on this dataset using 3-fold cross-validation.

### 2.3 Data processing of training maps

The grid size of the maps was unified to 1.0 Å by applying trilinear interpolation. For training, the ground truth secondary structure of each voxel was assigned according to the closest backbone atom (N, C or C$\alpha $ atom) within 3.0 Å, which was annotated using STRIDE [45]. Residues with structure codes of H, G or I were labeled as $\alpha $-helices (Helix). Residues with codes of B/b or E were labeled as $\beta $-strands (Sheet). The rest residues were labeled as Others. All the unassigned voxels (no backbone atom within 3.0 Å) were marked and excluded from training. Namely, the unassigned voxels will not contribute to the loss during training. The input density chunks of EMNUSS were of size 40 $\times $ 40 $\times $ 40, and the output predicted chunks of EMNUSS were of the same size. The input density maps (and their corresponding annotated ground truth maps) were cut into overlapping boxes of size 60 $\times $ 60 $\times $ 60 with a slide stride of 30. Density values of each map were clipped to be equal or greater than 0.0, and then normalized in each box to the range 0–1.0 by the maximum density value of each box. To ensure effective training, boxes that have $\ge $95% unassigned voxels were excluded from training.

### 2.4 Network training

Different EMNUSS models were trained for the datasets with different resolution ranges and all the training parameters were the same for different models. In total, 10% of the training maps were used as validation set during network training. Afterwards, all the maps from the training set and validation set were cut into boxes of size 60 $\times $60 $\times $ 60. The input training data were augmented through random 90${}^{\circ }$ rotations and by randomly cropping the input 60 $\times $ 60 $\times $ 60 box into a 40 $\times $ 40 $\times $ 40 chunk. The network was implemented with Pytorch. For each model, the network was trained for at most 300 epochs with 180 boxes employed in one batch. Adam optimizer was adopted to minimize the cross-entropy loss for the prediction. The loss is weighted by [0.25, 0.5, 0.25], which is roughly the reciprocal of the ratio for the numbers of voxels of three secondary structures (Helix, Sheet and Others) in the dataset. The initial learning rate was set to 1e-3, and no L2 regularization was applied ($weight\_decay=0$). Learning rate decay was adopted. Namely, the learning rate will be reduced to 1/2 of its current value if the average loss on training set does not descend for 4 epochs. The training procedure will be stopped when the learning rate reaches a minimum value of 1e-5. The model with the least validation loss was used in evaluation.

**Table 1:** Average voxel F1 scores and residue Q3 accuracies of EMNUSS, Emap2sec and Haruspex on the simulated map test set for different secondary structure classes. The maps are simulated from the PDB structures at two different resolutions: 6.0 and 10.0 Å

Metric	Method	6.0 Å				10.0 Å			
		Overall	Helix	Sheet	Others	Overall	Helix	Sheet	Others
Voxel F1 score	**EMNUSS**	**0.902**	**0.922**	**0.864**	**0.865**	**0.880**	**0.899**	**0.843**	**0.836**
	Emap2sec	0.796	0.844	0.755	0.713	0.757	0.791	0.729	0.664
	Haruspex	0.329	0.171	0.237	0.532	0.292	0.191	0.012	0.543
Residue Q3 accuracy	**EMNUSS**	**0.918**	**0.941**	**0.927**	0.863	**0.900**	**0.915**	**0.921**	0.838
	Emap2sec	0.831	0.866	0.866	0.718	0.798	0.843	0.839	0.681
	Haruspex	0.415	0.117	0.257	**0.885**	0.402	0.139	0.002	**0.944**

**Figure 2:**
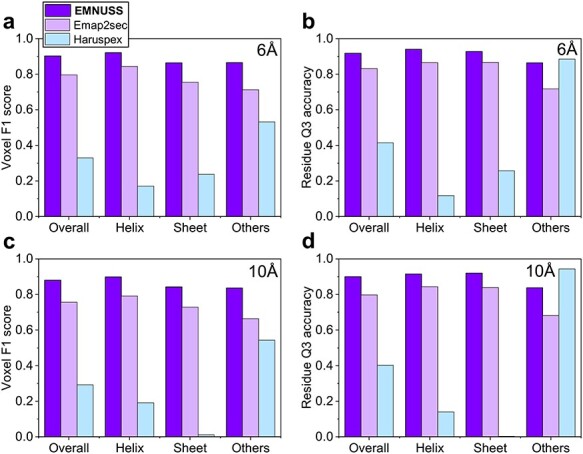
Comparison of EMNUSS, Emap2sec and Haruspex for different secondary structure classes on the simulated map test set, where the results of Emap2sec were taken from the literature [38]. (**A**) Average voxel F1 scores on the simulated maps at 6 Å. (**B**) Average residue Q3 accuracies on the simulated maps at 6 Å. (**C**) Average voxel F1 scores on the simulated maps at 10 Å. (**D**) Average residue Q3 accuracies on the simulated maps at 6 Å.

**Figure 3:**
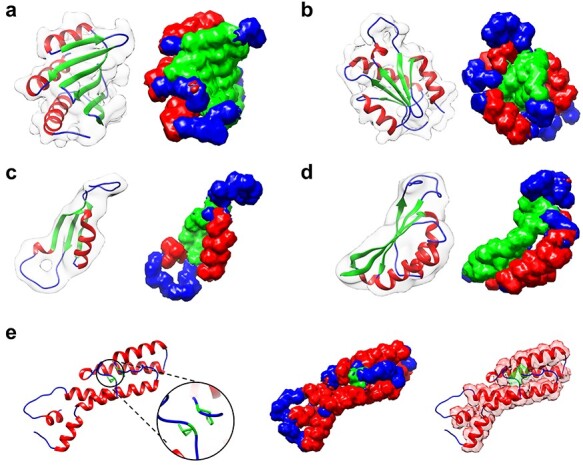
Examples of the secondary structure assignment by EMNUSS for the simulated maps at resolutions of 6.0 and 10.0 Å, where helices, strands and coils are colored in red, green and blue, respectively. The raw EM density map in transparent gray is overlapped with deposited PDB structure on the left side of each subfigure and the annotated map predicted by EMNUSS is on the right. (**A**) 6 Å simulated map for SCOPe entry d1kafa_. (**B**) 6 Å simulated map for SCOPe entry d1atia1. (**C**) 10 Å simulated map for SCOPe entry d1b33n_. (**D**) 10 Å simulated map for SCOPe entry d2cz4a1. (**E**) SCOPe entry d1a26a1, where the $\beta $-bridge structure (in black circle) is successfully detected by EMNUSS for 6 Å (middle) and 10 Å (right) simulated maps.

### 2.5 Evaluation of the EMUSS

We tested our EMNUSS on three different test sets. For each case in the test set, the EM density map was cut into boxes of size 60 $\times $ 60 $\times $ 60 with a slide stride of 30. Each box was normalized and then cropped into a 40 $\times $ 40 $\times $ 40 chunk from the center. Only the cube of size 30 $\times $ 30 $\times $ 30 at the center of each chunk was used to reconstruct the annotated map. In order to compare our results with Emap2sec, similar evaluation criteria were used. Secondary structure annotation by EMNUSS was evaluated at the voxel and amino acid residue levels. For each voxel in the map, the ground truth secondary structure was taken from the closest C$\alpha $ atom within 3.0 Å, which was defined by STRIDE annotation. The secondary structure of a C$\alpha $ atom was considered as correctly predicted if the majority of neighboring voxels that were within 3.0 Å of the atom have the correct secondary structure assignment. As the background in the EM map does not contain atomic information, we have also excluded those background voxels below a certain density threshold from the experimental maps. Therefore, each voxel in the EM map will have one of the three secondary structure classes (Helix, Sheet or Others) in our EMUSS model. To test the robustness of EMUSS, two different values of density thresholds were used in this study: 0.0 or the author-recommended contour level. A residue is defined to be below the threshold if any voxel around this residue (within 1.0 Å) has a density value below threshold. F1 score, which is the harmonic mean of the precision and recall of the assignments, was used to evaluate the performance of EMNUSS on voxels level. For the residue-level evaluation, residue Q3 accuracy was reported, which is the fraction of correctly assigned residues for the entire protein and for each of the three secondary structure classes.

### 2.6 Comparison with related works

EMNUSS was compared with Emap2sec on the test set of simulated maps and middle resolution maps. For the test set of simulated EM maps, we simply used the benchmarking values published in Emap2sec paper. For the test set of middle resolution EM maps, the trained models of Emap2sec taken from Code Ocean [46] were used. The input density data of Emap2sec were voxels of size 11 Å $\times $ 11 Å $\times $ 11 Å. The grid size of the maps was unified to 1.0 Å by applying trilinear interpolation of the electron density in the maps. The data were obtained from a map by traversing along the three dimensions of the voxels with a stride of 2.0 Å. As shown in Supplementary Table 2, compared with Phase 1, the Phase 2 network of Emap2sec performed worse, especially for those coil regions, such that in the following discussion we only compare EMNUSS to Phase 1 of Emap2sec.

EMNUSS was also compared with Haruspex on all three test sets. The Haruspex model was taken from the authors’ Github repository at https://github.com/thorn-lab/haruspex. The output of Haruspex was the annotated map of four channels representing codependent probabilities for four classes (‘helix’, ‘sheet’, ‘nucleotide’ or ‘unassigned’). Since nucleic acids were excluded from the test sets used in this study, the ’nucleotide’ channel of Haruspex was ignored in classification.

**Table 2:** Average voxel F1 scores and residue Q3 accuracies of EMNUSS, Emap2sec and Haruspex on the middle-resolution experimental map test set for different secondary structure classes. Two different values of threshold were used in evaluation: 0.0 or author-recommended contour level. All the voxels or residues below this threshold were excluded from evaluation

Metric	Method	Threshold = 0.0				Threshold = contour			
		Overall	Helix	Sheet	Others	Overall	Helix	Sheet	Others
Voxel F1 score	**EMNUSS**	**0.601**	**0.591**	**0.422**	**0.605**	**0.596**	**0.610**	**0.441**	**0.550**
	Emap2sec	0.331	0.249	0.229	0.395	0.480	0.492	0.361	0.429
	Haruspex	0.498	0.480	0.333	0.512	0.501	0.487	0.336	0.507
Residue Q3 accuracy	**EMNUSS**	**0.615**	0.576	**0.585**	**0.618**	**0.616**	**0.617**	**0.607**	**0.532**
	Emap2sec	0.319	0.148	0.419	0.438	0.507	0.480	0.574	0.459
	Haruspex	0.556	**0.582**	0.418	0.546	0.557	0.593	0.424	0.529

**Figure 4:**
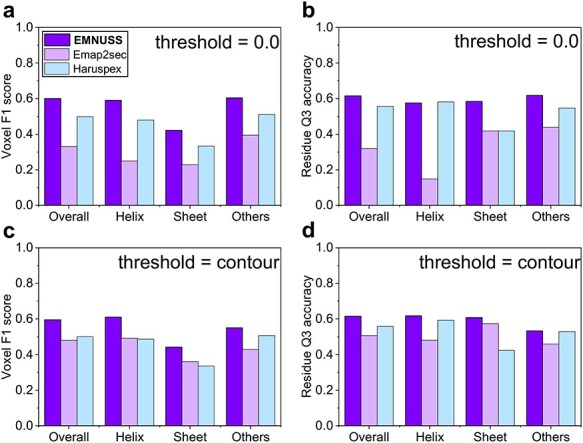
Comparison of EMNUSS, Emap2sec and Haruspex for different secondary structure classes on the middle-resolution experimental map test set, where the results of Emap2sec were directly calculated using the Emap2sec program [38, 47]. (**A**) Average voxel F1 scores using 0.0 as the threshold. (**B**) Average residue Q3 accuracies using 0.0 as the threshold. (**C**) Average voxel F1 scores using the author-recommended contour level as the threshold. (**D**) Average residue Q3 accuracies using the author recommended contour level as the threshold.

**Figure 5:**
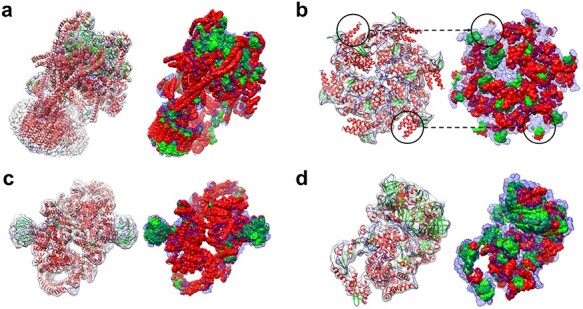
Examples of the secondary structure assignment by EMNUSS for middle-resolution experimental maps, where helices, strands and coils are colored in red, green and blue, respectively. The raw EM density map in transparent gray is overlapped with deposited PDB structure on the left, and the annotated map predicted by EMNUSS is on the right. Predicted coil regions (Others) are shown in transparency style. (**A**) EMD-8724 (PDB 5VOX). (**B**) EMD-8549 (PDB 5UIE). (**C**) EMD-3329 (PDB 5FVM). (**D**) EMD-3201 (PDB 5FKU).

## 3 Results and discussion

### 3.1 Secondary structure prediction for simulated maps

We first evaluated the performance of EMNUSS on the test set of 34 diverse SCOPe [43] protein domain structures. EM density maps are simulated from these structures at two different resolutions, 6.0 and 10.0 Å. Figure 2 shows a comparison of EMNUSS and Emap2sec in secondary structure detection at the voxel and residue levels. The corresponding benchmarking results are listed in Table 1. It can be seen from the figure that our EMNUSS significantly outperformed Emap2sec on the simulated map set. For the maps simulated at 6.0 Å, EMNUSS achieved an average overall voxel F1 score of 0.902, compared with 0.796 by Emap2sec (Figure 2**A**). The average voxel F1 scores for three different secondary structure classes were 0.922, 0.864 and 0.865 for Helix, Sheet and Others, respectively. At the residue level, EMNUSS also achieved a high-average overall residue Q3 accuracy of 0.918, which is much higher than 0.831 by Emap2sec. The average residue Q3 accuracies for three secondary structure classes were 0.941, 0.927 and 0.863 for Helix, Sheet and Others, respectively. Compared with the maps simulated at 6.0 Å, there was a slight decline in the performance of EMNUSS for the maps simulated at 10.0 Å (Figure 2**B**). An average overall voxel F1 score of 0.880 and an average overall residue Q3 accuracy of 0.900 were achieved by EMNUSS, which are still significantly higher than 0.757 and 0.798 achieved by Emap2sec. The average voxel F1 scores/residue Q3 accuracies for three different secondary structure classes were 0.899/0.915, 0.843/0.921 and 0.836/0.838 for Helix, Sheet and Others, respectively. We also evaluated the performance of Haruspex on this simulated map test set. However, Haruspex was designed for experimental EM maps and was not trained with simulated EM maps. Therefore, it did not work well on simulated maps. The overall voxel F1 score was only 0.329 and 0.292, and the overall residue Q3 accuracy was only 0.415 and 0.402, respectively, for simulated maps at 6.0 and 10.0 Å. Moreover, Haruspex has difficulties in detecting helices and sheets, resulting in low residue Q3 accuracies for helix and sheet and an exceptionally high residue Q3 accuracy for coil. The evaluation results for each map were listed in Supplementary Table 1.

Figure 3 shows several examples of secondary structure annotation by EMNUSS [47]. Figure 3**A** and **B** shows the results for the maps simulated at 6.0 Å. Figure 3**C** and **D** shows the results for the maps simulated at 10.0 Å. It can be seen from the figure that the predicted secondary structures shown at the right side agree with the ground truth secondary structure assignment shown at the left side very well. Interestingly, for SCOPe entry d1a26a1, the $\beta $-bridge structure between two residues ILE_691 and HIS_742 ignored by Emap2sec was correctly detected by EMNUSS (Figure 3**E**). These results demonstrated that our EMNUSS was indeed in capable of learning secondary structure information from EM maps.

**Table 3:** Average voxel F1 scores and residue Q3 accuracies of EMNUSS and Haruspex on the high-resolution experimental map test set for different secondary structure classes. Two different values of threshold were used in evaluation: 0.0 or author-recommended contour level. All the voxels or residues below this threshold were excluded from evaluation

Metric	Method	Threshold = 0.0				Threshold = contour			
		Overall	Helix	Sheet	Others	Overall	Helix	Sheet	Others
Voxel F1 score	**EMNUSS**	**0.838**	**0.860**	**0.731**	**0.791**	**0.846**	**0.878**	**0.755**	**0.775**
	Haruspex	0.595	0.590	0.487	0.561	0.612	0.624	0.515	0.553
Residue Q3 accuracy	**EMNUSS**	**0.852**	**0.876**	**0.826**	**0.779**	**0.861**	**0.893**	**0.849**	**0.754**
	Haruspex	0.741	0.843	0.576	0.659	0.746	0.892	0.652	0.582

### 3.2 Secondary structure prediction for middle-resolution experimental maps

Then, we compared EMNUSS with Emap2sec and Haruspex on the middle-resolution test set of 43 experimental EM maps with resolutions ranging from 5.0 to 9.5 Å. Two different values of thresholds were used: 0.0 or the author-recommended contour level. Figure 4 shows a comparison of the accuracies among the three methods at the voxel and residue levels. It can be seen from the figure that EMNUSS significantly outperformed Emap2sec and Haruspex on the middle-resolution experimental maps set. The corresponding benchmarking results are listed in Table 2. Using the author-recommended contour level as the threshold, EMNUSS achieved an average overall voxel F1 score of 0.596, compared with 0.480 by Emap2sec and 0.501 by Haruspex. At the residue level, EMNUSS achieved an average overall residue Q3 accuracy of 0.616, which is higher than 0.507 by Emap2sec and 0.557 by Haruspex. For individual secondary structure classes, EMNUSS also achieved higher voxel F1 scores and residue Q3 accuracies than Emap2sec and Haruspex. Although Haruspex showed a comparable performance with EMNUSS in predicting alpha helices in terms of residue Q3 accuracies, it performed substantially worse on predicting $\beta $-sheets and coils. See Supplementary Table 2 for details of the evaluation result of each map.

Another notable features is that there is only a little difference in performance for EMNUSS when choosing different threshold values. However, a sharp decline in performance was found for Emap2sec when using 0.0 as the threshold. It may be understood because Emap2sec filtered their training voxels according to the author-recommended contour level. In contrast, EMNUSS was trained with chunks that were cut from raw EM density maps, and thus, its solid performance was ensured on the entire map.

**Figure 6:**
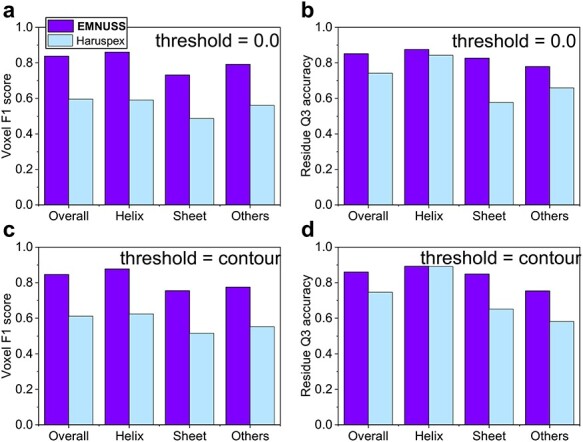
Comparison of EMNUSS and Haruspex for different secondary structure classes on the high-resolution experimental test set of 468 maps. (**A**) Average voxel F1 scores using 0.0 as the threshold. (**B**) Average residue Q3 accuracies using 0.0 as the threshold. (**C**) Average voxel F1 scores using the author-recommended contour level as the threshold. (**D**) Average residue Q3 accuracies using the author-recommended contour level as the threshold.

**Figure 7:**
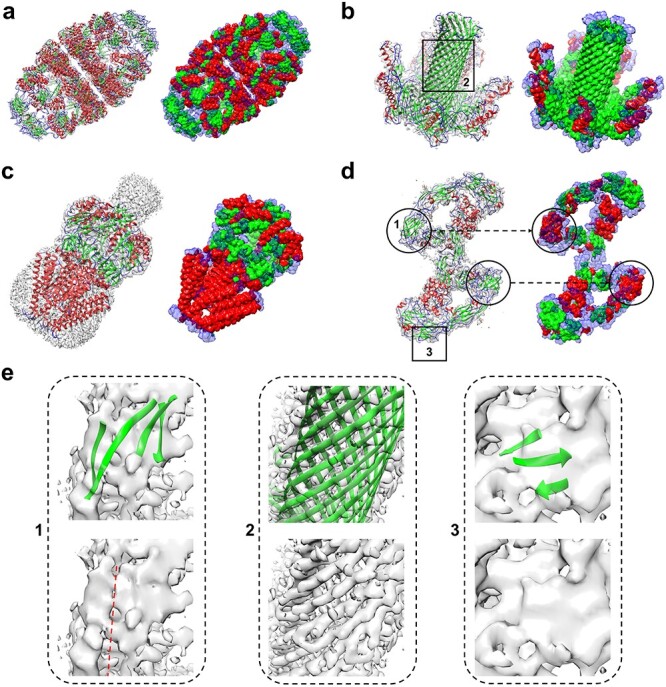
Examples of the secondary structure assignment by EMNUSS for high-resolution experimental maps, where helices, strands and coils are colored in red, green and blue, respectively. The raw EM density map in transparent gray is overlapped with the deposited PDB structure on the left, and the annotated map predicted by EMNUSS is on the right. Predicted coil regions (Others) are shown in transparency style. (A) EMD-9195 (PDB 6MRC). (B) EMD-4789 (PDB 6RB9). (C) EMD-4919 (PDB 6RLD). (D) EMD-20579 (PDB 6Q2S). (E) Enlarged view of $\beta $-sheet regions. Each subfigure corresponds to the region in black box with the same ID on (B) or (D). The density volume of $\beta $-sheet 1 is broken along the red dotted line.

**Figure 8:**
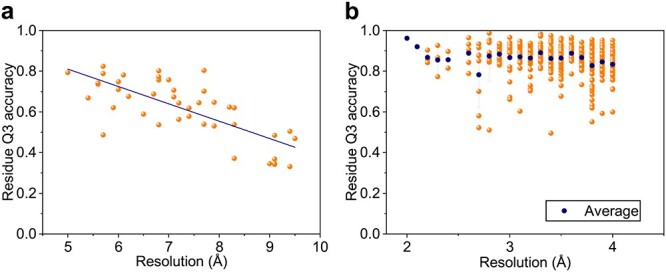
Residue Q3 accuracies of EMNUSS at different map resolutions. (A) The middle-resolution set, where the linear regression line is in navy blue. (B) The high-resolution set, where the average value for each resolution is shown in navy blue symbol, and error bars depict $\pm $1 SD.

Figure 5 shows several examples of secondary structure annotation by EMNUSS for the experimental EM maps at middle resolution. EMNUSS performed well on EMD-8724, a 6.8 Å map for the yeast V-ATPase in complex with *Legionella pneumophila* effector SidK [48], as illustrated in Figure 5**A**. EMNUSS successfully predicted most of the helix and strand regions, and thus yielded a high overall residue Q3 accuracy of 0.789 (0.801) when using 0.0 (contour) as the threshold. Figure 5**B** shows the results of EMD-8549, which is a 5.7 Å map for Vps4–Vta1 complex [49]. EMNUSS successfully predicted most of the helix and strand regions except for the peripheral helices that have relatively weak and fuzzy electron density signals in the EM map. The overall residue Q3 accuracy was 0.709 by using 0.0 as the threshold and increased to 0.824 after excluding those peripheral helices from evaluation by using contour as the threshold. For EMD-3329 in Figure 5**C**, which is a 6.1 Å map for the complex of Tor and Lst8 [50], EMNUSS successfully predicted the helix region in the middle and the $\beta $-strand-rich region on both sides and achieved high overall residue Q3 accuracies of 0.780 and 782 by using 0.0 and contour as the thresholds, respectively. Figure 5**D** gives the example of EMD-3201, which is an 8.3 Å map for *E. coli* replicative DNA polymerase complex in DNA-free state [51]. EMNUSS successfully detected the overall architecture of two $\beta $ subunits on the top. However, due to the poor resolution, EMNUSS crossed the borderline between helices and strands for the other subunits, resulted in a lower overall residue Q3 accuracy of 0.612 (0.620) using 0.0 (contour) as the threshold, compared with the above three examples.

### 3.3 Secondary structure prediction for high-resolution experimental maps

We further compared EMNUSS with Haruspex on the high-resolution dataset of 468 experimental maps with resolutions ranging from 2.0 to 4.0 Å. The training and testing of EMNUSS were performed on this dataset using 3-fold cross-validation. Figure 6 shows the performance of EMNUSS and Haruspex at the voxel and residue levels. The corresponding benchmarking results are listed in Table 3. It can be seen from the figure that EMNUSS performed significantly better than Haruspex on the high-resolution dataset. When using 0.0/author-recommended contour level as the threshold, EMNUSS achieved a high average overall voxel F1 score of 0.838/0.846, compared with 0.595/0.612 for Haruspex. At the residue level, EMNUSS also achieved a higher average overall residue Q3 accuracy of 0.852 and 0.861 when using 0.0 and contour as the thresholds, compared with 0.741 and 0.746 for Haruspex, respectively. Similar to the situation in middle-resolution test set, when using residue Q3 accuracies as the criteria, Haruspex showed a comparable performance with EMNUSS on predicting alpha helices but performed substantially worse than EMNUSS on predicting $\beta $-sheets and coils. The significantly higher voxel F1 scores for all three secondary structure classes achieved by EMNUSS suggests the better performance of EMNUSS than Haruspex. See Supplementary Table 4 for details of the evaluation results of each map.

Figure 7 shows several examples of secondary structure annotation by EMNUSS for the experimental EM maps at resolutions ranging from 2.0 to 4.0 Å. Figure 7**A** shows the results of EMD-9195, which is a 3.1 Å map for ADP-bound human mitochondrial Hsp60–Hsp10 football complex [52]. EMNUSS accurately annotated most of the helix and strand regions on this example, and thus yielded a high overall residue Q3 accuracy of 0.906 (0.923) when using 0.0 (contour) as the threshold. A $\beta $-strand-rich example EMD-4789 is displayed in Figure 7**B**, which is a 3.2 Å map for the pore structure of *Clostridium perfringens* epsilon toxin [53]. The core $\beta $-barrel region and peripheral helices were precisely annotated by EMNUSS. The overall residue Q3 accuracies were 0.853 and 0.868 when using 0.0 and contour as the threshold, respectively. Figure 7**C** gives the example of EMD-4919, which is a 2.9 Å map for mechanosensitive channel MSCS embedded in the membrane bilayer [54]. Although the transmembrane helices region (at the bottom) was coated with lipid bilayer in the EM map, EMNUSS successfully predicted most of the helix and strand regions and achieved a high overall residue Q3 accuracy of 0.932 (0.935) when using 0.0 (contour) as the threshold. One of the worst predictions made by EMNUSS is EMD-20579, which is a 3.8 Å cryo-EM map for RET/GFRa3/ARTN extracellular complex [55] (Figure 7**D**). EMNUSS achieved overall residue Q3 accuracies of 0.656 (0.659) when using 0.0 (contour) as the threshold on this example. As shown in the figure, EMNUSS yielded correct secondary structure annotation for almost the whole map except for the $\beta $-sheet-rich CLD4 domains marked by black circles. One of the possible reasons for such wrong prediction is that the electron density volumes around the $\beta $-sheets differ from typical density volumes of $\beta $-sheets. As illustrated in Figure 7**E**1, the density volume for the sheet is not completely connected. Thus, EMNUSS mistook the $\beta $-sheet for parallel helices. Figure 7**E**2 is an enlarged view of the $\beta $-barrel region in Figure 7**B**. The parallel $\beta $-strands can be seen clearly from the density volume. However, high-resolution map is indeed not essential for EMNUSS to make right predictions. As shown in Figure 7**E**3, another $\beta $-sheet region was successfully predicted for EMD-20579. Although individual $\beta $-strands are not visible here, the electron density signals of individual $\beta $-strands form an integrated surface, which is one of the typical electron density signals for a $\beta $-sheet. The essential reason of the incorrect predictions on such atypical density volumes is that the network is somehow short sighted and not acquainted with maps at lower resolutions. Thus, in order to improve the performance of EMNUSS, on one hand, the receptive field of EMNUSS should be broadened by optimizing the network architecture. On the other hand, a more detailed division of training maps according to resolutions is a potential way to improve the performance of EMNUSS.

### 3.4 Impact of map resolution

We further explored the impact of map resolution on the accuracy of EMNUSS. Since there was no significant difference in the performance of EMNUSS for choosing different threshold values, we only discussed the results using the author-recommended contour level as the threshold in this section.

Figure 8**A** shows the overall residue Q3 accuracy relative to the map resolution on the middle-resolution set. It can be seen from the figure that the performance of EMNUSS depends on the resolution. The Pearson correlation coefficient between residue Q3 accuracy and resolution was -0.746. Figure 8**B** shows the overall residue Q3 accuracy relative to the map resolution on high-resolution set. The performance of EMNUSS seems to have no significant correlation with resolution on the high-resolution set. The Pearson correlation coefficient between residue Q3 accuracy and resolution was only -0.138. Comparing Figure 8**A** and Figure 8**B**, we can find that the performance of EMNUSS has a significantly lower correlation with map resolution on the high-resolution dataset than on the middle-resolution set. Considering the fact that we have 300+ maps (in each cross-validation round) as the training set for high-resolution dataset compared with only 120 maps for middle-resolution dataset, this phenomenon may be attributed to the fact that EMNUSS was sufficiently trained on the high-resolution dataset. On the contrary, in order to improve the performance and robustness for the maps at middle resolution, more maps are needed for training.

**Figure 9:**
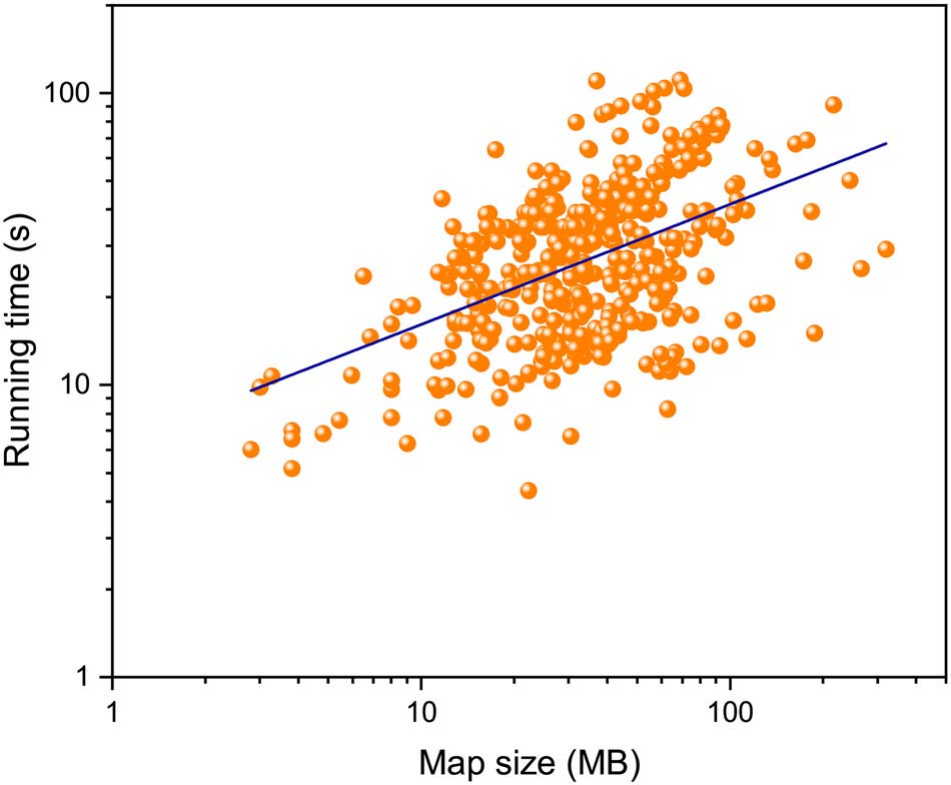
Running time of EMNUSS as a function of the map size on the high-resolution set in double logarithmic coordinates, where the power–law function regression line is shown in navy blue.

### 3.5 Running time

In addition to the high accuracy of EMNUSS, another preeminent character of EMNUSS is its high computational efficiency. We have measured the running times of EMNUSS for different sizes of input maps on the test set of 468 high-resolution experimental maps. On a NVIDIA Tesla K80 GPU device with 12 GB graphic memory, EMNUSS can finish a prediction for any test case in no more than 2 min. The average running time of EMNUSS was only 31.3 s, which is significantly faster than 70.1 s for Haruspex. Figure 9 shows that there is a power–law relationship between the running time of EMNUSS and the input map size. The relationship can be fitted with the following formula:

(1) \begin{align*}& t = 6.241 x^{0.412} \end{align*}

where $t$ is the expected running time in seconds for a map of size $x$ MB. Therefore, the running time for EMNUSS will increase much slower than the map size does, which is especially encouraging for large-size maps. For example, for EMNUSS, a 20 MB map takes about 20 s, and a 200 MB map only costs about 1 min. The running time–map size relationship can be understood because a larger map may have a large proportion of backgrounds that can be ignored by EMNUSS. See Supplementary Table 4 for the running times of each map.

## 4 Conclusion

We developed a deep learning framework to annotate protein secondary structures in EM density maps, which is referred to as EMNUSS. EMNUSS was extensively evaluated on three diverse test sets of simulated maps, middle-resolution experimental maps and high-resolution experimental maps. It was shown that EMNUSS significantly improved the accuracy of secondary structure detection and outperformed the existing approaches. Given its high efficiency and accuracy, it is anticipated that EMNUSS will serve as a valuable tool for secondary structure annotation for cryo-EM maps and help determine atomic structures for EM maps. With the increasing number of deposited EM density maps, the performance of EMNUSS can be further improved. One of our possible subsequent works of EMNUSS is to adding it into structural modeling for EM maps.

**Key Points**

- Secondary structure information is valuable for the atomic structure determination from cryo-EM maps.
- A novel deep learning framework was proposed to detect protein secondary structures in EM density maps.
- Our model adopts a 3D nested UNet architecture for fast and accurate prediction.
- Our model significantly outperformed existing approaches on both simulated and experimental EM maps.
- Our model is robust to handle cryo-EM maps of various sizes at both intermediate and high resolutions.

## Supplementary Material

Supplementary_Table_1_bbab156

Supplementary_Table_2_bbab156

Supplementary_Table_3_bbab156

Supplementary_Table_4_bbab156

## Data Availability

The EMNUSS program is available at http://huanglab.phys.hust.edu.cn/EMNUSS. The raw data of the secondary structure annotation results are provided in the Supplementary Information. The data that support the findings of this study are available from the corresponding author upon request.

## Notes

**Jiahua He** is a PhD student in the School of Physics at Huazhong University of Science and Technology. His research interests include molecular docking and protein structure prediction.

**Sheng-You Huang** is a full professor in the School of Physics at the Huazhong University of Science and Technology. His research interests are molecular docking and scoring functions for protein–ligand interactions, protein–protein interactions and protein–peptide interactions. More information about his research group can be found at http://huanglab.phys.hust.edu.cn.
